# Spontaneous regression of lung metastases in hepatocellular carcinoma: A case report

**DOI:** 10.1016/j.ijscr.2020.12.045

**Published:** 2020-12-24

**Authors:** Daisuke Muroya, Toshihiro Sato, Hisamune Sakai, Toru Hisaka, Yoshito Akagi, Koji Okuda

**Affiliations:** Department of Surgery, Kurume University, 67 Asahimachi, Kurume, Fukuoka Prefecture, 830-0003, Japan

**Keywords:** Hepatocellular carcinoma, Lung metastasis, Spontaneous regression

## Abstract

**Introduction:**

Spontaneous regression of hepatocellular carcinoma (HCC) is a rare condition. However, although there have been multiple reports of spontaneous regression, the definitive pathogenic mechanism of this phenomenon is still unclear.

**Case presentation:**

We encountered a case of a 78-year-old man who was undergoing dialysis for end-stage kidney disease with hepatitis C virus-associated chronic hepatitis presenting with HCC. The patient had previously undergone right lobectomy of the liver, but the cancer recurred with multiple lung metastases after 5 months. Approximately 13 months after the initial diagnosis of recurrence, the lung metastases decreased in size and eventually resolved without any anticancer therapy. The patient remains alive for over 41 months after recurrence.

**Discussion:**

Based on our case and literature, Hypoxia with hypotension due to hemodialysis can reduce the blood and oxygen supply of the body, which may lead to the spontaneous regression of the metastatic tumors.

**Conclusion:**

We herein reported a case of spontaneous regression of HCC undergoing dialysis.

## Highlights

• The prognosis of patients with advanced HCC remains poor even if appropriate treatments are administered.
• Spontaneous regression of lung metastases of hepatocellular is a rare condition.
• We discuss the mechanism for spontaneous regression of multiple pulmonary recurrences of hepatocellular carcinoma.

## 1 Introduction

Hepatocellular carcinoma (HCC) is one of the most common cancers and the third most common cause of cancer-related death worldwide. The prognosis of patients with advanced HCC remains poor even if appropriate treatments are administered. Patients with distant metastasis usually die within 12 months [1]. Spontaneous regression of HCC is a well-established phenomenon since 1972 [2]. This is defined as a partial or complete disappearance of the tumor without receiving any specific treatment [3] and is a very rare phenomenon with an incidence of 0.4% [4]. Furthermore, spontaneous regression of distant metastasis is a far rarer event [5,6]. Although there have been multiple reports of spontaneous regression, the definitive pathogenic mechanism of this phenomenon is still unclear. We present a case of resected HCC in a patient undergoing dialysis who exhibited spontaneous regression of the postoperative multiple lung metastasis without receiving any treatment and achieved long-term survival without progression.

This case report has been reported in line with the SCARE Criteria [include citation]’ at the end of the introductory section [7].

## 2 Case presentation

**Fig. 1:**
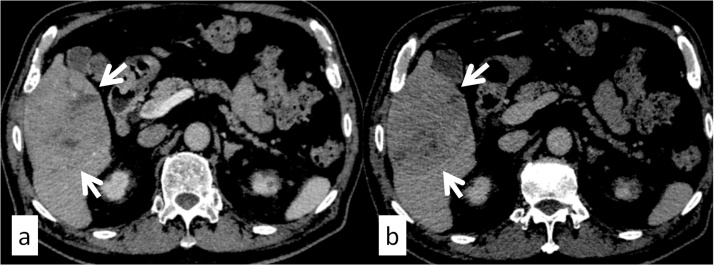
Preoperative computed tomography. A mass in the right lobe of the liver measuring 9.2 × 7.2 cm (white arrow), (a) demonstrating arterial hyperenhancement and (b) washout on delayed images; findings diagnostic of hepatocellular carcinoma.

**Fig. 2:**
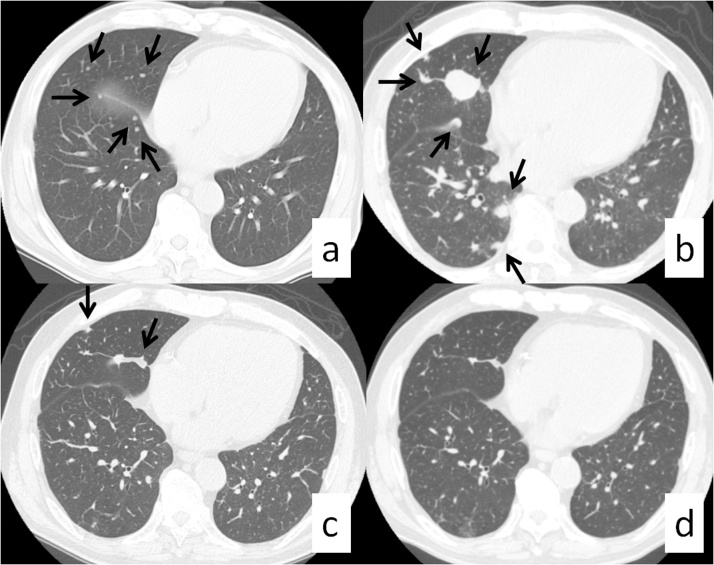
(a) CT chest revealed innumerable lung nodules concerning for metastatic disease. (b)The follow-up chest CT scan revealed progression of the lung tumor 13 months after the first presentation. (c)The follow-up chest CT scan revealed spontaneous regression of the lung tumor 18 months after the first presentation. (d) Almost complete disappearance of the lung metastasis 41 months after the first presentation (arrow).

**Fig. 3:**
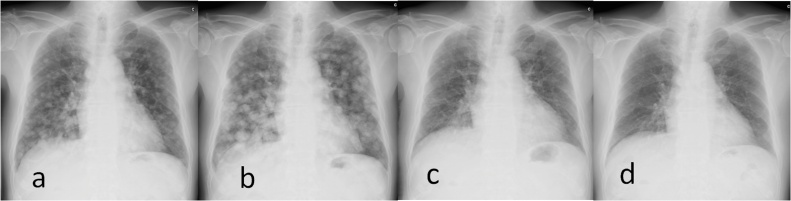
(a) Chest radiography showing innumerable lung nodules indicating metastatic disease 4 months after hepatectomy. (b) The follow-up chest radiography showing progression of the lung tumor 13 months after the first presentation. (c) The follow-up chest radiography showing spontaneous regression of the lung tumor 18 months after the first presentation. (d) Almost complete disappearance of the lung metastasis 41 months after the first presentation.

**Fig. 4:**
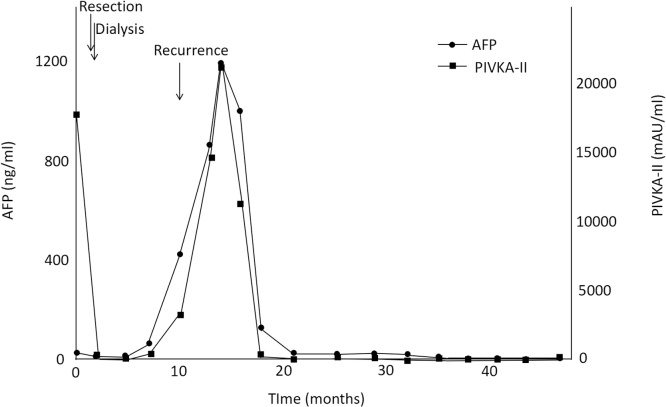
Clinical course of the levels of AFP and PIVKA-II. AFP, alpha fetoprotein; PIVKA-II, protein induced by vitamin K absence/antagonist-II.

A 78-year-old man with a history of hepatitis C and surgical resection of HCC was followed up for end-stage renal disease (ESRD) due to diabetes. The patient’s medical history included hepatic steatosis, hypertension, diabetes mellitus type 2 and ESRD. He had been prescribed metformin, pantoprazole, enalapril, felodipine, metoprolol. The patient was a non-smoker and non-drinker. At the age of 74 years, the patient was introduced to our hospital for HCC surgery (Fig. 1). His physical examination on admission was unremarkable. Liver enzymes and function tests, namely bilirubin (0.44 mg/dL), albumin (4.0 mg/dL), and International Normalized Ratio (1.06) were within the normal range, while creatinine was slightly elevated at 1.74 mg/dL. The patient underwent right lobectomy of the liver for HCC, and dialysis was initiated after surgery. At 5 months after the hepatic resection, computed tomography and chest radiography revealed multiple tumors in the lung, indicating metastatic disease (Fig. 2). At that time, serum protein induced by vitamin K absence or antagonist II (PIVKA-II) was abnormally elevated (648 mAU/mL). The serum alpha fetoprotein (AFP) level was 5.7 ng/mL. The patient did not receive any anticancer therapy because of his ESRD and the advanced stage of HCC. We subsequently followed up on his natural course with serial imaging. Chest radiography revealed an increase in the size and number of lung metastases, and tumor markers were significantly elevated 4 months after recurrence (AFP, 1198 ng/mL; PIVKA-II, 214155 mAU/mL). However, 13 months after recurrence, the metastatic lesions suddenly decreased in size and number without receiving any specific treatment or herbal medicine (Fig. 3). Follow-up imaging showed no evidence of disease progression, and tumor makers were significantly decreased (AFP, <1.0 ng/mL; PIVKA-II, 46 mAU/mL) (Fig. 4). There were no signs of new lesions on imaging or an increase in tumor markers over 41 months after the recurrence.

## 3 Discussion and conclusions

Spontaneous regression of cancer was first reported by Ole and Everson in 1956 [8], and the incidence rate was 1 out of 6000–10,000 cases [9]. Conventionally, this phenomenon is not uncommonly observed in malignant melanoma, neuroblastoma, and cancer of the kidney. However, in recent reports, the incidence of spontaneous regression in lung and liver cancers was also considered to be not rare. Based on the current literature review, many factors resulting in spontaneous regression of HCC have been proposed (Table 1) [[9], [10], [11], [12], [13], [14], [15], [16], [17], [18], [19], [20], [21], [22], [23], [24], [25], [26], [27], [28], [29], [30], [31], [32], [33], [34], [35], [36]]. Two common mechanisms of spontaneous HCC regression were identified: tumor hypoxia and systemic inflammatory and immunological activation [31,37].

**Table 1:** The mechanism of spontaneous regression of hepatocellular carcinoma.

tumor hypoxia	systemic inflammatory and immunological activation
Tumor thrombosis of hepatic artery [[9], [10], [11]]	Abstinence from alcohol [[23], [24], [25]]
Tumor thrombosis of portal vein [9,10,12]	Abstinence from smoking [23]
Hepatic angiography [13]	Herbal medicine [5,26]
Tumor rapid growth [[14], [15], [16]]	Prolonged fever [[27], [28], [29]]
Hepatic arterioportal shunts [17]	Antidiabetics [30]
Massive gastrointestinal hemorrhage [18,19]	Vitamin K administration [31]
Hemodialysis [3,20]	Bacterial infection [32]
Surgical invasion [21]	Abscopal effect of radiation [[33], [34], [35]]
Portal vein ligation [22]	

Tumor hypoxia is induced by the occlusion of the portal vein or feeding artery to the tumors, rapid tumor growth, large arterioportal shunt, chronic hypotension, and shock due to massive gastrointestinal bleeding [12,37,38]. The neoplastic tissue is more sensitive than the normal tissue to a sudden reduction in the blood and oxygen supply because of its high metabolic requirements [20]. However, our case did not have any of these pathophysiological conditions and clinical events, except for hemodialysis. Harimoto et al. [6] suggested that dialysis might play a role in the regression of HCC. In hemodialysis, there is a tendency for blood pressure to fluctuate both during and between hemodialysis treatments. Dialysis can reduce the blood and oxygen supply of the body, which may lead to the spontaneous regression of the metastatic tumors [6,39]. In the current case, the patient had started dialysis after surgery without taking any herbal medicine or consumed any new drugs. There were no symptoms suggesting a systemic inflammatory response including cholangitis, sepsis, and trauma [40,41]. The patient received no specific anticancer therapy nor had any known factors that affected tumor regression. Therefore, the spontaneous regression of HCC presented in this report may be attributed to the hypotension present during dialysis. Additionally, diabetes, the most common cause of chronic renal failure, also leads to hypotonia because of its systemic complications such as autonomic and peripheral neuropathy, macroangiopathy, and dynamic progression of atherosclerosis [42]. Although hypoxia with hypotension due to hemodialysis may be associated with the spontaneous regression of HCC, it cannot be concluded that only the dialysis affected the regression of HCC in the present case. In conclusion, we described a case of spontaneous regression of HCC undergoing dialysis. Further discussion and studies are needed to identify the mechanisms of this phenomenon, especially in association with dialysis.

## Declaration of Competing Interest

The authors report no declarations of interest.

## Funding

The authors declare that they have no funding for this research.

## Ethical approval

Not applicable.

## Consent

Written informed consent was obtained from the patient for publication of this case report and accompanying images.

## Author’s contribution

Daisuke Muroya: Investigation, Writing, Supervision, Hisamune Sakai: Resources, Toru Hisaka: Resources, Yoshito Akagi: Project administration, Koji Okuda: Writing- Reviewing and Editing.

## Registration of research studies

Not applicable.

## Guarantor

Professor Koji Okuda.

## Provenance and peer review

Not commissioned, externally peer-reviewed.
